# Population genetics and origin of horticultural germplasm in *Clematis* via genotyping-by-sequencing

**DOI:** 10.1093/hr/uhae336

**Published:** 2024-11-29

**Authors:** Yaping Hu, Qingdi Hu, Xiaohua Ma, Xule Zhang, Jian Zheng

**Affiliations:** Key Laboratory of Plant Innovation and Utilization, Institute of Subtropical Crops of Zhejiang Province, 324 Xueshan Road, Wenzhou 325005, China; Key Laboratory of Plant Innovation and Utilization, Institute of Subtropical Crops of Zhejiang Province, 324 Xueshan Road, Wenzhou 325005, China; Key Laboratory of Plant Innovation and Utilization, Institute of Subtropical Crops of Zhejiang Province, 324 Xueshan Road, Wenzhou 325005, China; Key Laboratory of Plant Innovation and Utilization, Institute of Subtropical Crops of Zhejiang Province, 324 Xueshan Road, Wenzhou 325005, China; Key Laboratory of Plant Innovation and Utilization, Institute of Subtropical Crops of Zhejiang Province, 324 Xueshan Road, Wenzhou 325005, China

Dear editor,

Genetic diversity serves as a cornerstone of the viability of a species within its natural habitat and constitutes the fundamental basis for genetic enhancement in cultivars [1, 2]. The genus *Clematis*, a member of the family Ranunculaceae, is a widely cultivated ornamental plant celebrated for its ornate flowers and varied morphological features. With a global distribution, *Clematis* includes >300 species, along with >3000 horticultural cultivars [3], manifesting pronounced genetic diversity. Owing to the lack of a reference genome for *Clematis*, studies on its genetic diversity have primarily relied on molecular marker techniques [4–6]. Genotyping-by-sequencing (GBS) can be used for the *de novo* genotyping of breeding panels and for developing accurate genomic selection models, even for large, complex, and polyploid genomes [7]. To date, no large-scale studies beyond traditional molecular marker techniques comprehensively explore the genetic background of *Clematis*.

Here, we selected 103 distinct *Clematis* samples from the entire collected germplasm, 35 of which were wild species, and the remaining were horticultural varieties. We used GBS to explore the genetic diversity and structure of *Clematis*. A collection of 103 *Clematis* accessions was successfully sequenced, yielding 3 284 011 564 raw reads, of which 3 270 742 974 (99.6%) were good barcode reads. After alignment and filtering, we identified 16 086 958 variants, of which 15 447 353 were single-nucleotide polymorphisms and the remaining were InDels, with an average density of one variant/1.98 kb (Fig. 1b). The most likely subpopulation classification for all *Clematis* cultivars was *K* = 2 after running STRUCTURE, starting with *K* = 1 (Fig. 1a). When *K* exceeded 2, the cross-validation error increased gradually. Consequently, we divided all 103 varieties into two subpopulations, designated C1 and C2, containing 25 and 78 varieties, respectively. Genetic structure was further investigated using principal component analysis, which provided non-parametric support for the aforementioned results. The first two principal components accounted for ~27.43% of the total variance, with PC1 and PC2 accounting for 14.29% and 13.14%, respectively. The tree revealed two major clades, corresponding to the genetic subpopulations identified in our earlier genetic structural analysis (Fig. 1c). Clades C1 and C2 include 25 and 78 samples, respectively. The separation of these two distinct clades suggests significant genetic divergence within the *Clematis* samples.

**Figure 1:**
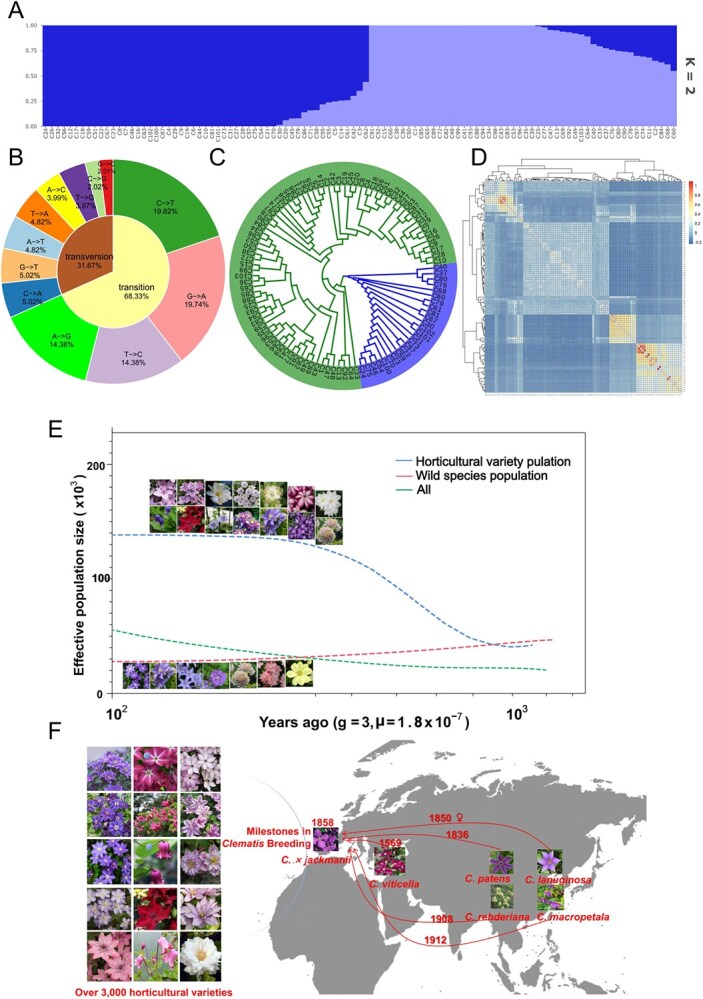
Genetic differences among *Clematis* populations. (A) ADMIXTURE analysis results for K = 2. (B) SNP conversion transversion types and ratios in *Clematis* genome sequencing. (C) Neighbor-joining tree of 103 *Clematis* samples generated from 1000 bootstrap replicates. (D) Heat map of estimated pairwise identity by descent values among *Clematis* samples*.* (E) Estimated historical effective population size of different populations. (F) Timeline depicting the trajectory of *Clematis* cultivation and development.

The kinship heat map of the 103 *Clematis* samples offered a comprehensive visualization of the genetic relationships within this diverse collection (Fig. 1d). Samples C17 and C9, C10 and C28, and C13 and C87 formed a tight cluster, indicating that they shared a significant amount of genetic material and likely had a common ancestry or had undergone similar selective breeding processes. Most of the sample pairs with identity by descent values >0.4 were combinations of wild species and horticultural varieties. In contrast, areas in the heat map are present where lighter colors, predominantly light blue, are more prevalent. These regions suggest lower genetic similarity between certain pairs of samples, highlighting the genetic diversity within the *Clematis* collection. This variation is essential for the genetic robustness of the collection and provides a broad genetic base for future breeding programs aimed at developing new varieties with desirable traits.

The effective number of alleles (Ne) for all 103 *Clematis* samples was 1.13121, with the C2 subpopulation showing a slightly higher Ne value (1.3253) than the C1 subpopulation (1.2661). A comparison of these values with the number of observed alleles (Na) suggested that the alleles in the C1 subpopulation were more evenly distributed across the population. For the entire *Clematis* population, the polymorphism information content (PIC) was calculated to be 0.1871, with the C1 and C2 subpopulations at 0.1544 and 0.1788, respectively. These values, all <0.25, suggested that the *Clematis* population exhibited low levels of polymorphism and genetic variation. This is consistent with previous genetic diversity studies of certain *Clematis* species using simple sequence repeats (SSR) and inter-simple sequence repeats molecular markers [4, 5]. Analysis of molecular variance (AMOVA) was conducted to examine genetic variation within and between *Clematis* populations, further elucidating the primary sources of variation. The results indicated that most genetic variation (99.02%) occurred within populations, whereas only 0.98% of the variation was attributed to differences between populations. This emphasizes that most of the genetic diversity in *Clematis* is maintained within individual subpopulations rather than between them. Genetic disparity between *Clematis* horticultural cultivars and wild species was considerably greater than that observed between subpopulations C1 and C2. The analysis revealed that the genetic diversity within the *Clematis* horticultural cultivars was higher than that of the wild populations. Extensive artificial cross-pollination among *Clematis* horticultural cultivars not only contributes to the creation of new varieties, but also enhances its genetic diversity. The evolutionary history of crop and horticultural plant varieties is marked by interspecific hybridization conducted by breeders during the creation of modern varieties. The phenotypic and genetic diversity of cultivated varieties arises not only from the combinations of alleles already present in the genomes of parental species but also from new genomic and epigenomic variations that emerge in first-generation hybrids, which have profound implications for the genetic diversity of populations [8]. The deliberate use of hybridization in plant breeding for domestication is intended to harness temporary hybrid vigor, generate desired variations between lineages, and produce new phenotypes [9, 10]. This is exemplified in >3000 horticultural varieties of *Clematis*.

Furthermore, we can explore the historical dynamics of *Clematis* horticultural varieties compared with wild populations by using SMC++. The results revealed that the effective population size of horticultural varieties began to increase gradually ~800 years ago. In contrast, the effective population size of the wild populations slowly decreased (Fig. 1e). The effective size of the entire *Clematis* population remained stable and then gradually increased, which was related to the increasing number of horticultural varieties. *Clematis* has a long history of cultivation, beginning in ancient China and Japan. By 1569, *Clematis viticella* from southeastern Europe had been cultivated and became a progenitor of many large-flowered varieties. In Britain, *Clematis vitalba* has been semicultivated for several centuries. The introduction of *Clematis patens* in China in 1836 and *Clematis lanuginosa* in Britain in 1850 sparked significant interest. These species have been cross-pollinated with *C. viticella*, initiating breeding programs in France and Britain, resulting in numerous large-flowered varieties. A major milestone was achieved in 1858 with the British cultivation of *C. × jackmanii*, a hybrid with *C. lanuginosa* as the maternal parent and mixed pollen from *Clematis eriostemon* and *C. viticella* ‘Atroruben’ (Fig. 1f).

Using the GBS data, we searched for all tags and identified 13 215 SSRs. We further compared these SSR results with the InDel results, yielding 2398 polymorphic SSRs. From these 2398 polymorphic SSRs, we randomly selected 30 SSRs and amplified them from the DNA of 30 *Clematis* germplasms. Nine pairs of primers exhibited high polymorphism, with an average polymorphic band percentage of 98.26%. The observed number of Na was 1.1204, that of Ne was 1.2149, Nei’s gene diversity (H) was 0.1785, and the Shannon information index (I) was 0.2895. The comparative results demonstrate that these nine pairs of primers can reveal high polymorphisms within *Clematis* germplasm resources, making them suitable for subsequent molecular marker applications.

## Data Availability

The high-throughput sequencing data in this study are open access and uploaded to the National Center for Biotechnology Information (NCBI) under accession number PRJNA1094651.
